# Supplementary material for: Increased antioxidative defense and reduced advanced glycation end-product formation by metabolic adaptation in non-small-cell-lung-cancer patients
Source: Nat Commun. 2025 Jun 3;16:5157. doi: 10.1038/s41467-025-60326-y (PMC12134105; doi:10.1038/s41467-025-60326-y)
Supplement: Supplementary file 1 — Supplementary Information [file 41467_2025_60326_MOESM1_ESM.pdf]

Increased antioxidative defense and reduced advanced glycation end-product formation by metabolic adaptation in NSCLC patients

Tamara Tomin[1], Sophie Elisabeth Honeder[1], Laura Liesinger[1], Daniela Gremel[1], Bernhard Retzl[1], Joerg Lindenmann[2], Luka Brcic[3], Matthias Schittmayer[1]* and Ruth Birner-Gruenberger[1,4]*

*co-corresponding authors contact: matthias.schittmayer@tuwien.ac.at and ruth.birner-gruenberger@tuwien.ac.at

[1] Institute of Chemical Technologies and Analytics, TU Wien, Vienna, Austria

[2] Division of Thoracic and Hyperbaric Surgery, Medical University of Graz, Graz, Austria

[3] Department of Pathology, Medical University of Vienna, Austria

[4] Diagnostic and Research institute of Pathology, Medical University of Graz, Graz, Austria

Supplementary Information

Supplementary Figures

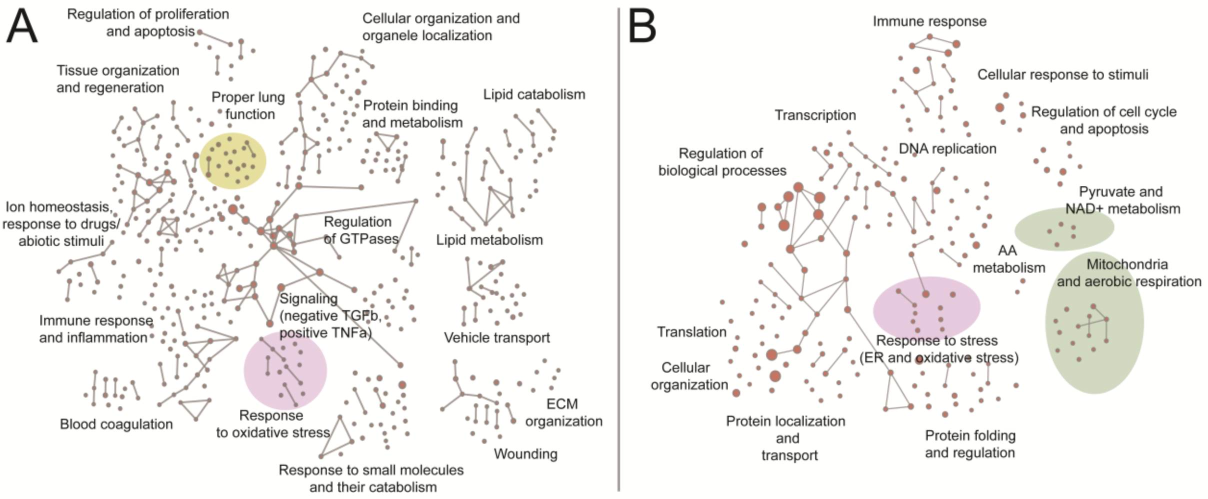

Figure S1. **Gene ontology enrichment of biological processes.** Significantly less (A) or significantly more (B) abundant proteins in tumor were used as input (threshold for protein to be considered significant: FDR corrected p-value < 0.05, S0 = 0.1; threshold for enrichment: FDR corrected p-value < 0.05; GO terms visualized with EnrichmentMap.

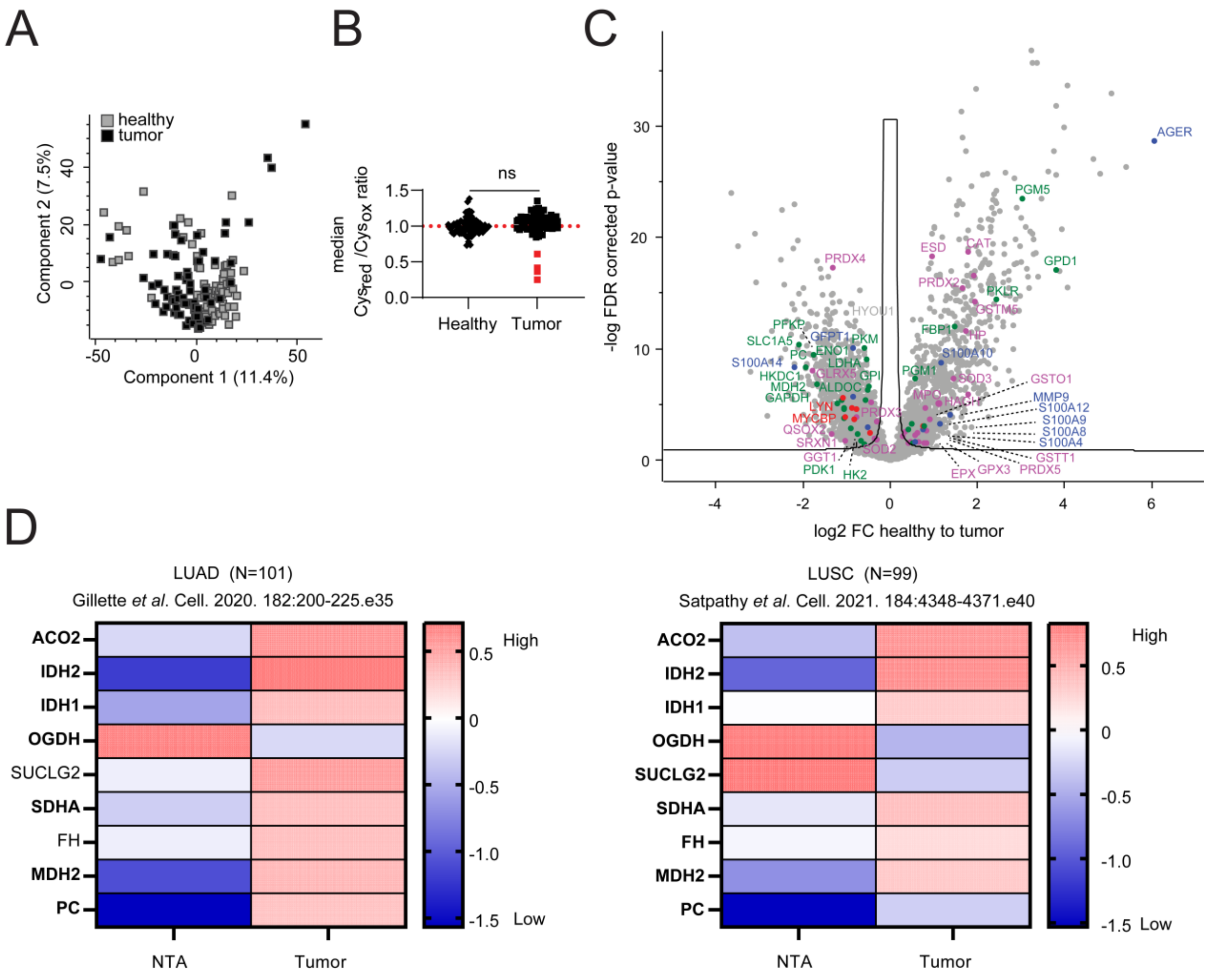

Figure S2. Redox and label free proteomics overview of the NSCLC patient lung tissue data. A. Principal component analysis of individual tumor and healthy samples in redox analysis with Cys_{red}/Cys_{ox} ratios as input. B. Together with PCA, median Cys_{red}/Cys_{ox} ratios between tumor and healthy tissue samples reveal minimal difference of the overall redox ratios between the two sample groups. C. Volcano plot of the LFQ proteomics data of the lung cancer patient samples (unpaired multi-test corrected two-sided Student's t-test p-value < 0.05, S0 = 0.1; green - glucose metabolism; pink - oxidative stress; blue - MG and advanced glycation endproducts, red - oncogenes). D. Target validation across two published pair-wise matched tumor vs. healthy proteomics datasets (left:lung adenocarcinoma (LUAD) dataset published in 2020 by Gillette *et al*. (N= 101 per group; tumor or non-tumor adjacent (NTA)[3]), right: lung squamous cell carcinoma dataset by Satpathy *et al*. published in 2021 (N=99 per group)[4]). Proteins marked in bold are significantly altered (p-value < 0,05) upon two-sided, paired Student t-test.

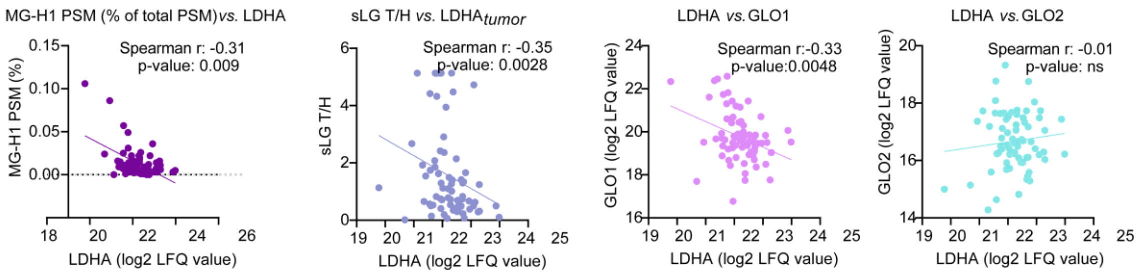

Figure S3. LDHA is negatively correlated with GLO1 abundance and MG-H1 modification frequency. Correlation analysis of tumor MG-H1 modification frequency (as % of all PSMs), s-lactoylglutathione (sLG) values (as tumor/healthy (T/H) ratio), tumor GLO1 and GLO2 protein levels with the abundance of LDHA. Numbers of pairs per correlation analysis: 69 (GLO2) - 70 (all other panels).

References

1. Tomin, T., Schittmayer, M. & Birner-Gruenberger, R. Addressing Glutathione Redox Status in Clinical Samples by Two-Step Alkylation with N-ethylmaleimide Isotopologues. *Metabolites* **10**, 71 (2020) doi: 10.3390/metabo10020071.

2. Tomin, T. *et al.* Mass Spectrometry-Based Redox and Protein Profiling of Failing Human Hearts. *IJMS* **22**, 1787 (2021) doi: 10.3390/ijms22041787.

3. Gillette, M. A. *et al.* Proteogenomic Characterization Reveals Therapeutic Vulnerabilities in Lung Adenocarcinoma. *Cell* **182**, 200-225.e35 (2020) doi: 10.1016/j.cell.2020.06.013.

4. Satpathy, S. *et al.* A proteogenomic portrait of lung squamous cell carcinoma. *Cell* **184**, 4348-4371.e40 (2021) doi: 10.1016/j.cell.2021.07.016.
