# Supplementary material for: Increased antioxidative defense and reduced advanced glycation end-product formation by metabolic adaptation in non-small-cell-lung-cancer patients
Source: Nat Commun. 2025 Jun 3;16:5157. doi: 10.1038/s41467-025-60326-y (PMC12134105; doi:10.1038/s41467-025-60326-y)
Supplement: Supplementary file 2 — Description of Additional Supplementary Information [file 41467_2025_60326_MOESM2_ESM.pdf]

Description of Additional Supplementary Information

Supplementary Data 1: patient metadata including smoking status, PET values and mutation states where available.

Supplementary Data 2: label free quantitative proteomics data from the patient cohort (N=70 patients, paired healthy vs. tumor tissue). Data included was subjected to two-sided, unpaired multi-testing corrected Student's t-tests. Significantly altered proteins marked.

Supplementary Data 3: redox proteomics data of the patient cohort (N=70 patients, paired healthy vs. tumor tissue). Included L/H ratios of cysteine residues were subjected to both multi-testing corrected as well as not corrected two-sided, unpaired Student's t-tests. Significantly altered redox ratios marked.

Supplementary Data 4: Subcellular localization analysis of significantly (uncorrected two-sided unpaired Student's t-test p-value < 0.05) more oxidized protein in tumor or healthy tissue as well as Gene ontology (GO) of biological processes with significantly redox altered proteins as input.

Supplementary Data 5: manually curated clusters of GOBP enrichment analysis with proteins significantly less abundant in tumor tissue (multi-testing corrected unpaired two-sided Student's t-test p-value < 0.05) as input for enrichment

Supplementary Data 6: manually curated clusters of GOBP enrichment analysis with proteins significantly more abundant in tumor tissue (multi-testing corrected unpaired two-sided Student's t-test p-value < 0.05) as input for enrichment.

Supplementary Data 7: Results from open and MG-H1 targeted search carried out on the patient data (N=70 patients, paired healthy vs. tumor tissue). MG-H1 targeted search represents a list of MG-H1 modified peptides and was subjected to filtering and multi-testing corrected, unpaired two-sided Student's t-tests.

Supplementary Data 8: Proteomics data from *in vitro*, cell culture experiments, including hypoxic exposure of H358 cells (N=4 biological replicates per time point and condition), GAPDH knock down in A549 and H358 cells (N=3 biological replicates per condition), as well as GAPDH inhibition in A549 and H359 cells (N=6 biological replicates per condition). All of the data was subjected to multi-test corrected, unpaired, two-sided Student t-tests.
