# Supplementary material for: Increased antioxidative defense and reduced advanced glycation end-product formation by metabolic adaptation in non-small-cell-lung-cancer patients
Source: Nat Commun. 2025 Jun 3;16:5157. doi: 10.1038/s41467-025-60326-y (PMC12134105; doi:10.1038/s41467-025-60326-y)
Supplement: Supplementary file 11 — Reporting Summary [file 41467_2025_60326_MOESM11_ESM.pdf]

nature portfolio

Corresponding author(s): Ruth Birner-Gruenberger
Matthias Schittmayer

Last updated by author(s): 14/5/25

Reporting Summary

Nature Portfolio wishes to improve the reproducibility of the work that we publish. This form provides structure for consistency and transparency in reporting. For further information on Nature Portfolio policies, see our Editorial Policies and the Editorial Policy Checklist.

Statistics

For all statistical analyses, confirm that the following items are present in the figure legend, table legend, main text, or Methods section.

n/a	Confirmed	
☐	☒	The exact sample size (*n*) for each experimental group/condition, given as a discrete number and unit of measurement
☐	☒	A statement on whether measurements were taken from distinct samples or whether the same sample was measured repeatedly
☐	☒	The statistical test(s) used AND whether they are one- or two-sided<br>*Only common tests should be described solely by name; describe more complex techniques in the Methods section.*
☒	☐	A description of all covariates tested
☐	☒	A description of any assumptions or corrections, such as tests of normality and adjustment for multiple comparisons
☐	☒	A full description of the statistical parameters including central tendency (e.g. means) or other basic estimates (e.g. regression coefficient) AND variation (e.g. standard deviation) or associated estimates of uncertainty (e.g. confidence intervals)
☐	☒	For null hypothesis testing, the test statistic (e.g. *F*, *t*, *r*) with confidence intervals, effect sizes, degrees of freedom and *P* value noted<br>*Give P values as exact values whenever suitable.*
☒	☐	For Bayesian analysis, information on the choice of priors and Markov chain Monte Carlo settings
☒	☐	For hierarchical and complex designs, identification of the appropriate level for tests and full reporting of outcomes
☐	☒	Estimates of effect sizes (e.g. Cohen's *d*, Pearson's *r*), indicating how they were calculated

Our web collection on statistics for biologists contains articles on many of the points above.

Software and code

Policy information about availability of computer code

Data collection	Mass spectrometry data was acquired with the corresponding manufacturer acquisition software: HyStar (v6.0.30.0; Bruker; proteomics data) and Realtime Analysis-LabSolutions (v5.97; Schimadzu; glutathione data). Western Blots were visualized with the integrated BioRad software (ChemiDoc).
Data analysis	Patient proteomics data analysis including database search and label free protein quantitation (LFQ) was carried out with MaxQuant (v2.1.3.0). Redox proteomics was processed with FragPipe (version 18.0 containing MSFragger v.3.5 and IonQuant 1.8.0) and consequently annotated with a Python script (https://github.com/BernhardRetzl/cysteine_label). Open search as well as targeted search for methylglyoxal modifications were also carried out with FragPipe (same version or 19.0 (containing MSFragger 3.6 and IonQuant 1.8.9)). Glutathione analytes were integrated using Schimadzu quantitative Browser from LabSolutions (v5.97). Perseus (v1.6.14.0) was used for analysis of proteomics data and GraphPad (v10.2.3) for all other comparisments. Enrichment analysis was done in Cytoscape (v3.9.1) equipted with StringApp (v1.6.0) and EnrichmentMap (v3.3.1). In vitro proteomics data analysis was carried out in DIA-NN software (v1.9.2).

For manuscripts utilizing custom algorithms or software that are central to the research but not yet described in published literature, software must be made available to editors and reviewers. We strongly encourage code deposition in a community repository (e.g. GitHub). See the Nature Portfolio guidelines for submitting code & software for further information.

Data

Policy information about availability of data

All manuscripts must include a data availability statement. This statement should provide the following information, where applicable:

- Accession codes, unique identifiers, or web links for publicly available datasets
- A description of any restrictions on data availability
- For clinical datasets or third party data, please ensure that the statement adheres to our policy

All patient raw proteomics data including search engine output has been deposited to PRIDE with the following identification: PXD052340.
The code for cysteine position annotation and calculation of unique redox ratios is available via GitHub (https://github.com/BernhardRetzl/cysteine_label)
In vitro proteomics data (hypoxia) was submitted to PRIDE under the following credentials: PXD062503.
In vitro proteomics data (GAPDH inhibition) was submitted to PRIDE under following credentials: PXD061610.

Research involving human participants, their data, or biological material

Policy information about studies with human participants or human data. See also policy information about sex, gender (identity/presentation), and sexual orientation and race, ethnicity and racism.

Reporting on sex and gender	The patient cohort (n=70) consisted of 64 % male and 36 % female patients. There was no gender-based separation or gender-specific analysis.
Reporting on race, ethnicity, or other socially relevant groupings	N/A
Population characteristics	Average age of the cohort was 65 ± 9 years at the point of surgery. For large majority (96 % of the patients) this surgery was the first line of treatment, rendering the cohort mainly chemo-naïve. The dominant form of NSCLC was lung adenocarcinoma (60 %) followed by squamous cell carcinoma (31 %). In 7 % of the cases no further pathological stratification at the point of sample collection beyond "NSCLC" was available or mixed pathology was reported. One case of large cell carcinoma was included.
Recruitment	Study included all consented patients who were undergoing curative surgery for confirmed NSCLC in 2019/2020 at the Division of Thoracic and Hyperbaric Surgery at the Medical University/State hospital of Graz (Austria).
Ethics oversight	The use of human biomaterials was approved by the ethics committee of the Medical University of Graz (30-354 ex 17/18).

Note that full information on the approval of the study protocol must also be provided in the manuscript.

Field-specific reporting

Please select the one below that is the best fit for your research. If you are not sure, read the appropriate sections before making your selection.

[x] Life sciences   [ ] Behavioural & social sciences   [ ] Ecological, evolutionary & environmental sciences

For a reference copy of the document with all sections, see nature.com/documents/nr-reporting-summary-flat.pdf

Life sciences study design

All studies must disclose on these points even when the disclosure is negative.

Sample size	Thee study includes 70 participants. No statistical analysis was carried out to determine sample size - it was dictated by sample availability over the given time span.
Data exclusions	Due to high biological variability no samples were excluded and samples from all 70 patients were used for proteomics, glutathione and s-lactoylglutathione analysis. Analysis of small thiols (glutathione precursors and degradation products) was carried out on 15 patients due to sample availability and no samples were excluded. In case of correlation of hypoxic markers with GLO1/2 abundance in H358 cells (Figure 4GH) one sample from 1% oxygen/48 h condition was excluded due to overall low protein quantitation values. The data is nevertheless reported in the Supplementary Data 8.
Replication	For patient samples, proteomics, glutathione and s-Lactoylglutathione analysis was carried out on 70 biological replicates per condition (tumor or healthy). Analysis of small thiols (glutathione precursors) was carried out on 15 patients due to sample availability. Cell experiments were carried out either on four replicates per condition and time point (hypoxia; 21 % or 1 % oxygen and 48 or 72 h, respectively); or three replicates per condition (siRNA control or siGAPDH; two different cell lines) or six replicates per condition (GAPDH inhibition; DMSO control or 10 µM koningic acid; two different cell lines). GAPDH western blot was repeated twice and corroborated by proteomics experiments.
Randomization	Patient samples were assigned ascending numbers upon collection (1 to 70) but were processed within a week at random order. For the LC-MS measurement, matched tumor and healthy samples were measured alternatively (one tumor sample, then one healthy sample)

	preventing any condition-related batch effects. In vitro experiments were processed at random group order and measured alternatively across different conditions, preventing any condition-specific batch effects.
Blinding	This study compares 2 conditions (tumor or healthy) of 70 participants in a single group, no blinding was applied to allow direct comparison of patient matched healthy and tumor tissue. For in vitro experiments also no blinding was applied.

Reporting for specific materials, systems and methods

We require information from authors about some types of materials, experimental systems and methods used in many studies. Here, indicate whether each material, system or method listed is relevant to your study. If you are not sure if a list item applies to your research, read the appropriate section before selecting a response.

Materials & experimental systems

n/a	Involved in the study
☐	☒ Antibodies
☐	☒ Eukaryotic cell lines
☒	☐ Palaeontology and archaeology
☒	☐ Animals and other organisms
☒	☐ Clinical data
☒	☐ Dual use research of concern
☒	☐ Plants

Methods

n/a	Involved in the study
☒	☐ ChIP-seq
☒	☐ Flow cytometry
☒	☐ MRI-based neuroimaging

Antibodies

Antibodies used	anti-GAPDH mouse monoclonal antibody (60004-1-IG; Proteintech)<br>anti-vinculin mouse monoclonal antibody (sc-73614; Santa Cruz)<br>HRP-linked goat anti-mouse secondary antibody (G21040; Invitrogen)
Validation	Both antibodies are commercial and were therefore validated both by the manufacturer as well as in the previous studies, including KO validation.<br>GAPDH: https://www.ptglab.com/products/GAPDH-Antibody-60004-1-Ig.htm?srsltid=AfmBOoqvpUrB2WDhW5_uVKWx346_u8Z1aht5L1pkI6f2Q1TXr8PC3WEF<br>vinculin: https://www.scbt.com/p/vinculin-antibody-7f9?srsltid=AfmBOoqlzFTmOonMFQiQFMzNM5LCoO2n8FUnJAwq9Mu-1oXCOzJ0CH7O<br>Secondary:<br>https://www.thermofisher.com/antibody/product/Goat-anti-Mouse-IgG-H-L-Cross-Adsorbed-Secondary-Antibody-Polyclonal/G-21040

Eukaryotic cell lines

Policy information about cell lines and Sex and Gender in Research

Cell line source(s)	Cell Lines Service (Eppelheim, Germany; A549 cells) or the American Type Culture Collection (ATCC, Manassas, VA, USA; H358 cells).
Authentication	STR profiling
Mycoplasma contamination	Cells were regularly tested for Mycoplasm using fluorescent kit (Lonza).
Commonly misidentified lines<br>(See ICLAC register)	N/A

Plants

Seed stocks	N/A
Novel plant genotypes	N/A
Authentication	N/A
